# Developing a modern data workflow for regularly updated data

**DOI:** 10.1371/journal.pbio.3000125

**Published:** 2019-01-29

**Authors:** Glenda M. Yenni, Erica M. Christensen, Ellen K. Bledsoe, Sarah R. Supp, Renata M. Diaz, Ethan P. White, S. K. Morgan Ernest

**Affiliations:** 1 Department of Wildlife Ecology and Conservation, University of Florida, Gainesville, Florida, United States of America; 2 School of Natural Resources and the Environment, University of Florida, Gainesville, Florida, United States of America; 3 Data Analytics Program, Denison University, Granville, Ohio, United States of America; 4 Informatics Institute, University of Florida, Gainesville, Florida, United States of America; 5 Biodiversity Institute, University of Florida, Gainesville, Florida, United States of America

## Abstract

Over the past decade, biology has undergone a data revolution in how researchers collect data and the amount of data being collected. An emerging challenge that has received limited attention in biology is managing, working with, and providing access to data under continual active collection. Regularly updated data present unique challenges in quality assurance and control, data publication, archiving, and reproducibility. We developed a workflow for a long-term ecological study that addresses many of the challenges associated with managing this type of data. We do this by leveraging existing tools to 1) perform quality assurance and control; 2) import, restructure, version, and archive data; 3) rapidly publish new data in ways that ensure appropriate credit to all contributors; and 4) automate most steps in the data pipeline to reduce the time and effort required by researchers. The workflow leverages tools from software development, including version control and continuous integration, to create a modern data management system that automates the pipeline.

## Abstract 2

This Community Page article describes a data management workflow that can be readily implemented by small research teams and which solves the core challenges of managing regularly updating data. It includes a template repository and tutorial to assist others in setting up their own regularly updating data management systems.

## Introduction

Biology has transitioned from a field in which data are collected in hand-written notes by lone scientists to a discipline that increasingly involves large amounts of data collected by collaborative teams. The impact of the increased volume of data being collected has been extensively discussed in biology [1,2], but there has also been a revolution in the frequency with which data are collected [3]. Instead of one-time data collection, biologists are increasingly collecting data that require databases to be regularly updated with new information. Long-term observational studies, experiments with repeated sampling, use of automatic sensors, and ongoing literature mining to build data compilations all produce continually updating data. These data are being used to address problems that require regularly updating data streams, including adaptive monitoring and management [4], iterative near-term forecasting [5], detecting and preventing ecological transitions [6], and monitoring real-time cancer metabolism [7]. Thus, whether studying changes in gene expression over time or long-term population dynamics, data that are being analyzed while they are still undergoing data collection are becoming a pervasive aspect of biology.

Data that are frequently updated present unique challenges for effective data management, reproducibility, and credit. Regularly updated data (Box 1) requires continual data entry, data integration, and error checking. This need for continually active data management places an extra burden on researchers and increases the potential for delays between when data are collected and when they are available to analyze. Since the data are continually changing, it is also essential to have methods for tracking, comparing, and archiving different versions of the data to support reproducibility [1,8]. Finally, since new contributors often join ongoing projects, a method is needed that provides credit to new contributors while still allowing the impact of the project as a whole to be tracked. While strategies for managing large amounts of actively updated data exist in biology, they are typically limited to large, institutionalized data collection efforts with dedicated informatics groups. To reduce delays and burden on individual labs and small teams, researchers need accessible protocols that promote rapid, ongoing data entry, versioning, archiving, and documentation.

**Box 1. Terminology**

This regularly updated biological data differs from conventional “streaming data” in that it typically involves manually collected data, requires data entry, and is not truly continuous in nature. This type of data has been referred to by a variety of terms including “dynamic data” [37], “evolving data” [38,39,18], and “living data” [40], but there is no general consensus on the appropriate terminology. To communicate more effectively across these different groups, we chose to simply describe the key aspect of this data that makes it a challenge to work with: the fact that this is data under continuing data collection that results in frequent updating of data files.

As a small group of researchers managing an ongoing, long-term research project, we have grappled with the challenges of managing data that is regularly updated and making it publicly available. Our research involves automated and manual data collection efforts at daily to annual frequencies conducted over 40 years by a regularly changing group of personnel (Box 2; for details on our study and data collection see [9]). Thus, our experience covers much of the range of challenges that biologists are struggling to manage when their data are continually being updated. We designed a modern workflow system to expedite the management of data streams, ranging from hourly data collected by automated weather stations to plant and animal data recorded on datasheets in the field. We have designed our process to mitigate the data management workload by automating much of the data management pipeline. We use a variety of tools that range from those commonly used in biology (e.g., Microsoft Excel and programming in R) to tools that have primarily been used in only the highly computational areas of biological research (e.g., version control and continuous integration; for more information see S1 Box and S2 Box, respectively). We use these tools not only to help with the regular addition of new data but also to provide clear documentation when we find and fix existing errors in the database that evaded earlier quality assurance/quality control (QA/QC) procedures. Here, we describe our approach with the goal of allowing others to implement similar data management systems and to improve the data management of regularly updated data more broadly.

**Box 2. The model system for this paper**

Our data are generated by the Portal Project, a long-term study in ecology that is currently run by our research group [9]. The project was established by Dr. James H. Brown in 1977 in the southwestern United States to study competition among rodents and ants and the impact of these species on desert plants [41]. We collect data for several datasets that regularly update at different frequencies (hourly, monthly, biannually, and annually), and each data set presents its own challenges.

Low-frequency, sample unit–level plant data

We collect, on paper data sheets, information on the number of plant individuals per sampling quadrat but do not track particular individuals through time. These data are the least intensive to manage because data entry and quality control activities are more concentrated in time, and there are fewer potential issues for us to check.

High-frequency, individual-level rodent data

These data are time intensive to manage because they are recorded monthly on paper data sheets and require extra quality control efforts to maintain accurate individual-level data.

Highest-frequency, automated weather data

We also collect weather data, generated hourly, which we download weekly from an automated weather station at the field site. Because we do not transcribe these data, there are no human-introduced errors. We perform weekly quality control efforts for these data to detect any issues with the sensors, including checking for abnormal values and comparing output to regional stations to identify extreme deviations from regional conditions.

Given the variety of data that we collect, we require a generally flexible approach for managing the data coming from our study site. The diversity of regularly updating data that we manage makes it likely that our data workflow will address many of the data management situations that biologists collecting updating data regularly encounter.

## Implementing a modern data workflow

Setting up an automated data management system for regularly updated data may initially seem beyond the skill set of most empirically focused lab groups. The approach we have designed and describe below does require some level of familiarity with computational tools such as a programming language (e.g., Python or R) and a version control system (e.g., git). However, data management and programming are increasingly becoming core skills in biology [10], even for empirically focused lab groups, and training in the tools we used to build our data management system is available at many universities or through workshops at conferences. In designing and building the infrastructure for our study, our group consisted primarily of field ecologists who received their training in this manner and sought assistance from a computational ecologist for help with design and implementation of some of the more advanced aspects. We have aimed this paper and our associated tutorial at empirical groups with little background in the tools or approaches we implemented. Our goal is to provide an introduction to the concepts and tools, general information on how such a system can be constructed, and assistance—through a tutorial—for building data management systems to manage regularly updating data. Box 3 contains a recipe for implementing our approach. Readers can also peruse our active data repository (www.github.com/weecology/PortalData) to see details of how we constructed our pipeline.

**Box 3. Recipe for creating a regularly updating data pipeline**

Visit https://www.updatingdata.org/ for click-through instructions on how to follow this recipe to build a regularly updating data repository.

1. Clone the livedat repository
2. Configure the repository for your project
3. Connect to Zenodo
4. Connect to Travis
5. Give Travis access to update your data on GitHub
6. Add data
7. Add quality assurance/quality control (QA/QC) code
8. Update data

### QA in data entry

**Fig 1:**
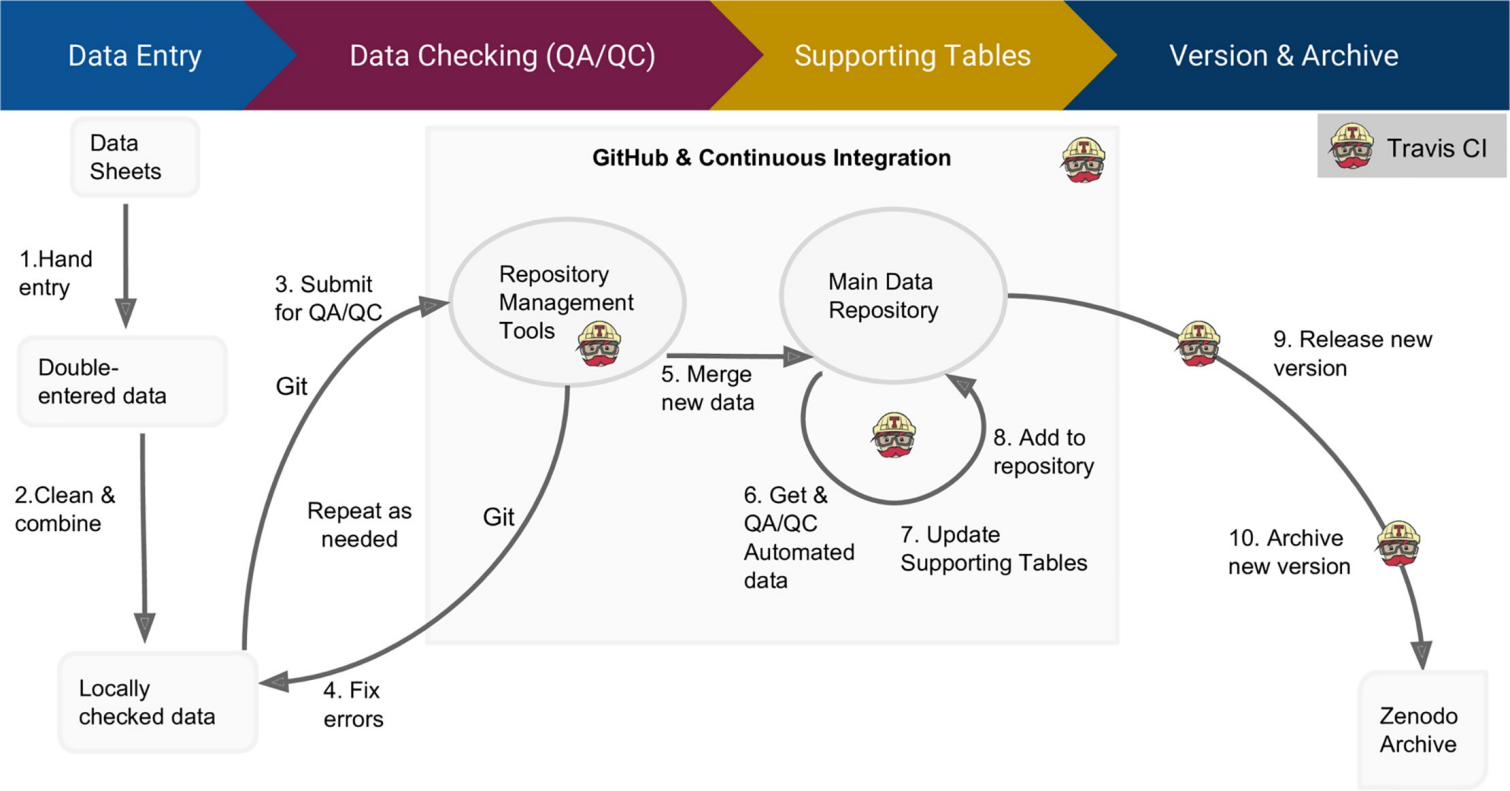
Data workflow for regularly updated data. 1. All field-collected data are double entered with automated checks to prevent invalid values from being entered. 2. The two versions of the double-entered data are compared using an R script, and mismatches are corrected. 3. A pull request is submitted to the data repository (i.e., GitHub), which triggers data checks run by the continuous integration system (i.e., Travis CI). 4. If the system detects any issues, the update is reviewed again, and corrections are made to the pull request, automatically triggering the data checks to run again. 5. Once the new data pass all automated checks, a data manager reviews the changes and merges the new data into the main data repository. 6. Addition of the new data triggers the continuous integration system to run additional scripts to get data from automated sensors (e.g., weather data) and to check for errors. 7. The system then runs scripts that automatically update the supporting tables (information not collected in the field that helps with data use) and updates the version number. 8. Once all tables have been automatically updated, the updates are automatically merged into the main repository. 9. The system automatically triggers a new release on GitHub. 10. The GitHub–Zenodo integration sends the new data release as a new version to Zenodo for archiving. CI, continuous integration.

For data collected onto data sheets in the field or the lab, the initial processing requires human interaction to enter the data. Using tools and approaches that automatically check data for errors can make this process more efficient. Upon returning from the field, two different people manually enter the new data into Excel spreadsheets (Fig 1, step 1). We use the “data validation” feature in Excel to restrict possible entries as an initial method of quality control by restricting accepted species codes to those on a prespecified list and defining allowable ranges for numeric values. The two separately entered versions are compared to each other using an R script to find errors from data entry (Fig 1, step 2). The R script detects any discrepancies between the two versions and returns a list of row numbers in the spreadsheet where these discrepancies occur, which the researcher then uses to compare to the original data sheets and fix the errors.

### Adding data to the central database

When data are regularly updated, often by multiple researchers, it is essential to have a central version of the database with all of the most current data. We store our data in comma separated values (csv) files in a system designed for managing and tracking changes to files called version control. Version control was originally designed for tracking changes to code but can track changes to any digital file, including data files [11–13]. We use a specific version control system—git—and the associated GitHub website (Fig 1, “GitHub and Continuous Integration”) for managing version control (see S1 Box for more details). We store the master version of the data files online on GitHub (https://github.com/weecology/PortalData). The data, along with the code for data management, are stored in the version control equivalent of a folder, called a repository (Fig 1, “Main Data Repository”). Through this online repository, everyone in the project has access to the most up-to-date or “master” version of both the data and the data management code. To add or change data in this central repository, we create a copy of the repository on a user’s local computer, which we then edit, save any changes along with a message describing the changes, and send a request through GitHub (called a “pull request”; Fig 1, step 3) to have these changes integrated into the central repository (S1 Box). While any user can suggest a change or addition, only select individuals have the authority to merge these changes into the central version (see S1 Box). This version control based process retains records of every change made to the data along with an explanation of that change [11–13]. It also makes it possible to identify changes between different stages and to go back to any previous state of the data. As such, it protects data from accidental changes and makes it possible to track the provenance of the data.

### Automated data checks

Automating data checks (i.e., QA/QC) are essential for efficiently delivering regularly updated data of high quality. We automate a variety of aspects of our data management system, including the data checks, by using “continuous analysis” (sensu [14]), an approach for automating computational analyses. Continuous analysis uses “continuous integration” tools from software engineering to automatically run a set of commands (in our case, this includes R code that is run to error check new data) when data or code is updated or at user-specified times ([14]; see S2 Box). Continuous integration systems (we use Travis CI; https://travis-ci.com/) are designed to interact with version control systems, which makes it relatively easy to automate QA/QC checks of the data [15]. When a “pull request” to add new data to the central database is submitted, it automatically triggers the continuous integration system to run a predetermined set of QA/QC checks. The QA/QC checks the validity and consistency of the new data (e.g., do data for all samples exist, are data values that should be similar through time self-consistent). This QA/QC system uses a software-testing approach called “unit testing” that is typically used to check that pieces of code work in the expected way [16]. We use tests, written using the “testthat” package, to do our unit testing [17]. Any identified issues with the data are automatically flagged in the pull request, indicating that they need to be fixed before the data are added to the main repository (Fig 1, step 4). The researcher then identifies the proper fix for the issue, fixes it in their local copy, and updates the pull request, which is then automatically retested to ensure that the data pass QA/QC (Fig 1, step 3).

### Human review and updating the central database

Human review of data updates is useful for identifying issues that are difficult to detect programmatically. Before field data are merged into the main repository, we require human review of the proposed changes by someone other than the researcher who initiated the pull request. This review is facilitated by the pull request functionality on GitHub, which shows the reviewer only the lines of data that have been changed [11]. Once the changes have passed both the automated tests and human review, a data manager confirms the merge, and the changes are incorporated into the main version of the database (Fig 1, step 5). Records of all merged pull requests are retained in git and on GitHub, and it is possible to revert to previous states of the data at any time.

### Automatically integrating data from sensors

Many data collection efforts in biology involve some sort of automated data collection. We collect hourly weather data from an on-site weather station that transmits data over a cellular connection; we also download data from other weather stations in the region whose data are streamed online. While data collected by automated sensors do not require steps to correct human-entry errors, they still require QA/QC for sensor errors, and the raw data need to be processed into the most appropriate form for our database. To automate this process, we developed R scripts to download the data, transform them into the appropriate format, run QA/QC checks, and automatically update the weather table in the main repository (Fig 1, steps 6 and 8). The continuous integration system is scheduled to regularly download and add new weather data. Errors identified by the QA/QC checks will cause our continuous integration system to register an error, indicating that the data require human attention before being added to the main repository (similar to the QA/QC process described in Fig 1, steps 3 and 4). This process yields fully automated collection of weather data in near-real time.

### Automated updating of supporting tables

Once data from the field are merged into the main repository, there are often supporting data tables that need to be updated. Supporting tables contain information (e.g., about data collection events such as sampling intensity or timing) that cannot be efficiently stored in the main data file. Since this information can be derived from the entered data, we have automated the process of updating supporting tables in order to reduce the time and effort required to incorporate new sampling events into the database (Fig 1, steps 7 and 8). For each table that needs to be updated, we wrote a function to 1) confirm that the supporting table needs to be updated, 2) extract the relevant information from the new data in the main data table, 3) perform data checks, and 4) append the new information to the supporting table. The update process is triggered by the addition of new data into one of the main data tables, at which point the continuous integration service executes these functions (see S2 Box). Automating curation of these supporting tables reduces the potential for data entry errors and allows researchers to allocate their time and effort to tasks that require intellectual input.

### Versioning

A common issue with data sets that are regularly updated is that the data available at one point in time are not the same as the data at some point in the future, which can cause difficulties for reproducing and comparing analyses [11,13]. Creating distinct versions of the database every time it changes and timestamping those versions allows analyses that can be run on a specific version of the data [18,13]. To address this issue, we automatically make a “release” every time new data are added to the database (using the GitHub application programming interface [API]; Fig 1, step 9). This allows specific versions of the data used for an analysis to be referenced directly, and the exact form of the data can be downloaded to allow fully reproducible analyses even as the data set is continually updated. Versions are named following the newly developed Frictionless Data data-versioning guidelines (https://frictionlessdata.io/specs/patterns/; see [13] for a similar approach).

### Archiving

Satisfying journal and funding agency data requirements increasingly requires depositing data in an archive that guarantees stable long-term availability of data under an open license. GitHub repositories can be deleted at any time and therefore cannot serve as an archive [19,20]. Regularly updated data need an archive that supports easily automated archiving, data versioning, and DOIs for citation. In some fields, disciplinary repositories are the best choice for archiving some kinds of data, but often, these repositories do not support automatic updating. We archive our data with a Creative Commons 0 (CC0) license on Zenodo (https://zenodo.org/), a widely used general purpose repository, because it provides all of the necessary components. With its easy integration with GitHub, we can archive our data automatically with each update to the data (Fig 1, step 10; [13]). Zenodo’s data versioning provides DOIs that allow data users to cite both the exact version of the data used in their analyses (to allow for fully reproducible analyses) and the data set as a whole (to allow accurate tracking of the usage of the data set). To support the archiving of regularly updated data, data archives should support automatic updating (e.g., via an API) and data versioning.

### Citation and authorship

Regularly updating data also produces complexities for providing academic credit for collecting and sharing data, which is essential for a healthy culture supporting data collection and reuse [21,22]. Data papers, which allow a data set to be treated like a publication for reporting and citation, are modeled on scientific papers and are effectively static. This limits their utility when data is being added repeatedly over time because there is no established way to update the data, metadata, or authorship. The ideal solution is a data paper that can be updated to include new authors, mention new techniques, and link directly to continually updating data in a data repository. This would allow the content and authorship to remain up-to-date and allow citations to acknowledge the use of the data set as a whole. We have addressed this problem by writing a data paper [9] that resides on BioRxiv, a preprint server widely used in the biological sciences. The data paper can be updated with new versions as needed, providing the flexibility to add additional details, information on new data types, and new authors. BioRxiv supports versioning of preprints, which provides a record of changes to the data paper and to authorship. Citations to BioRxiv preprints are tracked by Google Scholar, providing academic credit that can be used to justify continued data collection to funders.

## Discussion

Data management and sharing are receiving increasing attention in science, resulting in new requirements from journals and funding agencies. Discussions about modern data management focus primarily on two main challenges: making data used in scientific papers available in useful formats to increase transparency and reproducibility [21,22] and the difficulties of working with exceptionally large data [23]. An emerging data management challenge that has received significantly less attention in biology is managing, working with, and providing access to data that are undergoing continual active collection. These data present unique challenges in quality assurance and control, data publication, archiving, and reproducibility. The workflow we developed for our long-term study solves many of the challenges of managing this type of regularly updating data. We employ a combination of existing tools to reduce data errors, import and restructure data, archive and version the data, and automate most steps in the data pipeline to reduce the time and effort required by researchers. This workflow expands the idea of continuous analysis (sensu [14]) to create a modern data management system that uses tools from software development to automate the data collection, processing, and publication pipeline.

We use our data management system to manage data collected both in the field by hand and automatically by machines, but our system is applicable to other types of data collection as well. For example, teams of scientists are increasingly interested in consolidating information scattered across publications and other sources into centralized databases, e.g., plant traits [24,25], tropical diseases [26], biodiversity time series [27], vertebrate endocrine levels [28], and microRNA target interactions [29]. Because new data are always being generated and published, literature compilations also have the potential for continual data expansion. Whether part of a large, international team such as the above efforts or single researchers interested in conducting meta-analyses, phylogenetic analyses, or compiling DNA reference libraries for barcodes, our approach is flexible enough to apply to most types of data collection activities for which data need to be ready for analysis before the endpoint is reached.

The main limitation on the infrastructure we have designed is that it cannot handle truly large data. Online services like GitHub and Travis CI typically limit the amount of storage and compute time that can be used by a single project. GitHub limits repository size to 1 GB and file size to 100 MB. As a result, remote sensing images, genomes, and other data types requiring large amounts of storage will not be suitable for the GitHub-centered approach outlined here. Travis CI limits the amount of time that code can run on its infrastructure for free to one hour. Most research data and data processing will fit comfortably within these limits (the largest file in the Portal database is currently <20 MB, and it takes <15 minutes for all data checking and processing code to run), so this type of system will work for the majority of research projects. However, in cases for which larger data files or longer run times are necessary, it is possible to adapt our general approach by using equivalent tools that can be run on local computing resources (e.g., GitLab for managing git repositories and Jenkins for continuous integration) and using tools that are designed for versioning large data (e.g., dat [30] or git Large File Storage [31]).

One advantage of our approach to the challenges of regularly updated data is that it can be accomplished by a small team composed of primarily empirical researchers. Our approach does not require dedicated information technology (IT) staff, but it does require some level of familiarity with tools that are not commonly used in biology. Many research groups will need computational training or assistance. The use of programming languages for data manipulation, whether in R, Python, or another language, is increasingly common, and many universities offer courses that teach the fundamentals of data science and data management (e.g., http://www.datacarpentry.org/semester-biology/). Training activities can also be found at many scientific society meetings and through workshops run by groups like the Carpentries, a nonprofit group focused on teaching data management and software skills—including git and GitHub—to scientists (https://carpentries.org/). A set of resources for learning the core skills and tools discussed in this paper is provided in S3 Box. The tool that is most difficult to learn is continuous integration, both because it is a more advanced computational skill not covered in most biology training courses and because existing documentation is primarily aimed at people with high levels of technical training (e.g., software developers). To help researchers implement this aspect of the workflow, including the automated releasing and archiving of data, we have created a starter repository including reusable code (http://github.com/weecology/livedat). Our website (https://www.updatingdata.org) provides a dynamic tutorial to help researchers set up continuous integration and automated archiving using Travis CI for their own GitHub repository. The value of the tools used here emphasizes the need for more computational training for scientists at all career stages, a widely recognized need in biology [32–34]. Given the importance of making continually collected data rapidly available for forecasting and other research, the field will continue to need to train, support, and retain scientists with advanced computational skills to assist with setting up and managing regularly updating data workflows.

The rise of technology to aid data collection in the sciences has fundamentally changed how we quantify and measure biological activities (e.g., [35]) and facilitates our ability to find and compile information from the literature and across systems. While the resulting ability to generate data sets that are regularly updated with new information will help address complex issues facing our society (e.g. climate change, emerging diseases, cancer prevention and treatment), it also comes with unique challenges. We have described some of these challenges and our approach to solving them in the hope that it can serve as a catalyst for future development to make implementing data management protocols for this type of data more broadly accessible. All stages of the workflow for regularly updated data (Fig 1) could be made easier to implement through improved tooling. A priority for investment in this area is simplifying the setup of continuous analysis systems for the data management–focused challenges of automated versioning and archiving. Additional training in automation and continuous analysis for biologists will also be important for helping the scientific community advance this new area of data management. There are also a number of important issues that, while not central to our project, need to be addressed to maximize the management of regularly updating data more generally. In particular, we see three areas to address: 1) data licensing issues for heterogeneous data sets (especially for data compilations [36]); 2) properly crediting contributions to tool development (e.g., software, data management pipelines), especially for early career researchers; and 3) determining standards for authorship for large distributed collaborations. Continually updated data will become an increasingly more common data type in biology. This makes investment now in the tools, training, and culture of dealing with continually updated data critical for ensuring that scientists can maximize their use of this emerging data type to address pressing questions in biology. See Box 4 for glossary of terms.

**Box 4. Glossary**

CI: Continuous integration (also see S2 Box). The continuous application of quality control. A practice used in software engineering to continuously implement processes for automated testing and integration of new code into a project.

Git: (also see S1 Box) Git is an open source program for tracking changes in text files (version control) and is the core technology of which GitHub, the social and user interface, is built on top.

GitHub: (also see S1 Box) A web-based hosting service for version control using git.

Github–Travis CI integration: Connects the Travis CI continuous integration service to build and test projects hosted at GitHub. Once set up, a GitHub project will automatically deploy CI and test pull requests through Travis CI.

Github–Zenodo integration: Connects a Github project to a Zenodo archive. Zenodo takes an archive of your GitHub repository each time you create a new release.

Pull request: A set of proposed changes to the files in a GitHub repository made by one collaborator, to be reviewed by other collaborators before being accepted or rejected.

QA/QC: Quality assurance/quality control. The process of ensuring the data in our repository meet a certain quality standard.

Repository: A location (folder) containing all of the files for a particular project. Files could include code, data files, or documentation. Each file’s revision history is also stored in the repository.

testthat: An R package that facilitates formal, automated testing.

Travis CI: (also see S2 Box) A hosted continuous integration service that is used to test and build GitHub projects. Open source projects are tested at no charge.

Unit test: A software-testing approach that checks to make sure that pieces of code work in the expected way.

Version control: A system for managing changes made to a file or set of files over time that allows the user to a) see what changes were made when and b) revert back to a previous state if desired.

Zenodo: A general, open-access, research data repository.

## Supporting information

S1 Box A brief introduction to version control using git and GitHub. (PDF)

S2 Box A brief introduction to continuous integration and an example setup using Travis. (PDF)

S3 Box Resources for learning data management tools. (PDF)

## Abbreviations

API: application programming interface
CC0: creative commons 0
CI: continuous integration
csv: comma separated values
IT: information technology
QA/QC: quality assurance/quality control
