# Supplementary material for: Developing a modern data workflow for regularly updated data
Source: PLoS Biol. 2019 Jan 29;17(1):e3000125. doi: 10.1371/journal.pbio.3000125 (PMC6368360; doi:10.1371/journal.pbio.3000125)
Supplement: S1 Box — (PDF) [file pbio.3000125.s001.pdf]

S1 Box: Version controlling data using git and Github

Version control systems are a set of tools for continually tracking and archiving changes made to a set of files. These systems were originally designed to facilitate collaborative work on software that was being continuously updated but can also be used when working with moderately sized data files. Version control tracks information about changes to files using “commits,” which record the precise changes made to a file or group of files along with a message describing why those changes were made. We use one of the most popular version control systems, git, along with an online system for managing shared git repositories, GitHub.

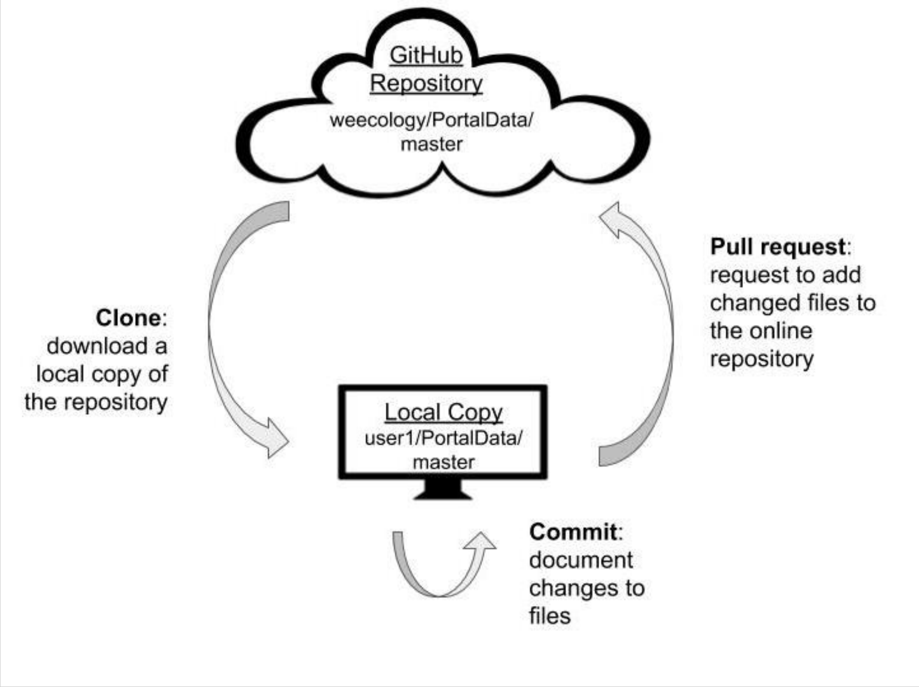

Version controlled projects are stored in “repositories,” (akin to a folder) and there is typically a central copy of the repository online to allow collaboration. In our case, this is our main GitHub repository that is considered to be the official version of the data (https://github.com/weecology/PortalData). Only approved users can edit this central repository directly, but any user can create their own copies of the main repository called “forks” or “clones”. Changes made to these copies do not automatically change the main copy of the repository. This allows users to have one or more copies of the master version where they can make and check changes (e.g., adding data, changing data-cleaning code) before they are added to the main repository. As the user makes changes to their copy of the repository, they document their work by “committing” their changes. The version control system maintains a record of each commit, and it is possible to revert to past states of the data at any time. Once a set of changes is complete, they can be “merged” into the main repository through a process called a “pull request”. A pull request is a request by a user for someone with administrator permissions on the repository an approved user or administrator to merge their changes into the main repository holding the primary copy of the data or code

(a request that your changes be “pulled” into the main repository). As part of the pull request process, Github highlights all of the changes from the master version (additions or deletions), making it easy to see what changes are being proposed and determine whether they are good changes to make. Pull requests can also be automatically tested to make sure that the proposed changes do not alter the core functionality of the code or the core requirements of the data. Once the pull request is accepted, those changes become part of the main repository, but can be undone at any time if needed.
