# Supplementary material for: Developing a modern data workflow for regularly updated data
Source: PLoS Biol. 2019 Jan 29;17(1):e3000125. doi: 10.1371/journal.pbio.3000125 (PMC6368360; doi:10.1371/journal.pbio.3000125)
Supplement: S2 Box — (PDF) [file pbio.3000125.s002.pdf]

S2 Box: Travis CI

Continuous integration is used in software engineering to automate testing and integrate new code into the main code base of a project. While designed as a software development tool, continuous integration has features which are useful for automating the management of updated data: it detects changes in files, automates running code, and tests output for consistency. Because these tasks are also useful in a research context, continuous analysis is also used to drive research pipelines (Beaulieu-Jones and Greene, 2017). We expand on this concept by applying continuous integration to the management of regularly updated data.

The continuous integration service that we use to manage our data is Travis CI (travis-ci.org), which integrates easily with Github. We tell Travis CI which tasks to perform by including a .travis.yml file (example below) in the GitHub repository containing our data, which is then executed whenever Travis CI is triggered.

Below is our .travis.yml file and how it specifies the tasks for Travis CI to perform. First, Travis CI runs an R script that will install all R packages listed in the script (the “install:” step). It then executes a series of R scripts that update tables and run QA/QC tests in the Portal Data repository (the “script:” step):

- Update the regional weather tables [line 10]
- Run the tests (using the testthat package) [line 11]
- Update the weather tables from our weather station [line 12]
- Update the rodent trapping table (if new rodent data have been added, this table will grow, otherwise it will stay the same) [line 13]
- Update the plots table (if new rodent data have been added, this table will grow, otherwise it will stay the same) [line 14]
- Update the new moons table (if new rodent data have been added, this table will grow, otherwise it will stay the same) [line 15]
- Update the plant census table (if new plant data have been added, this table will grow, otherwise it will stay the same) [line 16]
- Make sure Travis CI’s session is on the master branch of the repo [line 17]
- Run an R script to update the version of the data (see the versioning section for more details) and commit new changes to the master branch of the repository [line 18]

If any of the above scripts fail, the build will stop and return an error that will help users determine what is causing the failure.

Once all the above steps have successfully completed, Travis CI will perform a final series of tasks (the “after_success:” step). You can add additional steps here that will not break the build if they fail.

.travis.yml:

```
language: r
cache: packages
sudo: false
warnings_are_errors: false

install:
  - Rscript install-packages.R

script:
  - R -e 'setwd("DataCleaningScripts"); source("get_regional_weather.R"); append_regional_weather()'
  - Rscript testthat.R
  - R -e 'setwd("DataCleaningScripts"); source("new_weather_data.R"); append_weather()'
  - R -e 'setwd("DataCleaningScripts"); source("update_portal_rodent_trapping.r"); writetrappingtable()'
  - R -e 'setwd("DataCleaningScripts"); source("update_portal_plots.R"); writeportalplots()'
  - R -e 'setwd("DataCleaningScripts"); source("new_moon_numbers.r"); writenewmoons()'
  - R -e 'setwd("DataCleaningScripts"); source("update_portal_plant_censuses.R"); writecensustable()'
  - git checkout master
  - Rscript archive.R

after_success:
```

Travis CI not only runs on the main repository, but also runs its tests on pull requests before they are merged. This automates the QA/QC and allows detecting data issues before changes are made to the main datasets or code. If the pull request causes no errors when Travis CI runs it, it is ready for human review and merging with the repository. After merging, Travis CI runs again in the master branch, committing any changes to the data to the main database. Travis CI runs whenever pull requests are made or changes detected in the repository, but can also be scheduled to run automatically at time intervals specified by the user, a feature we use to download data from our automated weather station.
