# Supplementary material for: Developing a modern data workflow for regularly updated data
Source: PLoS Biol. 2019 Jan 29;17(1):e3000125. doi: 10.1371/journal.pbio.3000125 (PMC6368360; doi:10.1371/journal.pbio.3000125)
Supplement: S3 Box — (PDF) [file pbio.3000125.s003.pdf]

S3 Box: Resources for Learning Data Management Tools

It can be challenging to figure out how to get started or where to learn more about the tools we discuss. Here are some good places to find more information on specific aspects of our workflow for managing regularly updated data.

Get Started

Living data Starter Repository: Our template data repository, with a step-by-step setup guide. http://github.com/weecology/livedat

Living Data Setup Guide: A dynamic step-by-step guide to setting up your own data repository following our model, using the livedat repository as a template. https://www.updatingdata.org/

Open Source Licenses: Picking a license for your data. https://choosealicense.com/

Unit Testing: Information on how to construct tests. http://r-pkgs.had.co.nz/tests.html

Data Validation in Excel: Tutorial on how to restrict the types of values that Excel will accept during manual data entry. https://support.microsoft.com/en-us/help/211485/description-and-examples-of-data-validation-in-excel

Stack Overflow: A forum for asking and finding answers to your programming questions. https://stackoverflow.com/

Git/Git Hosts

Version Control for Beginners: A guide to the concept of version control. https://www.atlassian.com/git/tutorials

Resources to learn git: A resource page to help users learn Git, the most commonly-used version control system. https://try.github.io/

GitHub Learning Lab: A learning bot that will help users learn how to use GitHub to manage version control through practical exercises. Requires an account on GitHub (https://github.com) to use. https://lab.github.com/

Learn Git with Bitbucket: Bitbucket is an alternative to GitHub which also hosts git repositories. https://www.atlassian.com/git/tutorials/learn-git-with-bitbucket-cloud

Get Started with GitLab: GitLab is another alternative to GitHub that also hosts git repositories. https://docs.gitlab.com/ee/intro/

GitHub-Zenodo Integration: Learn more about the GitHub-Zenodo integration we use to automate archiving. https://guides.github.com/activities/citable-code/

Continuous Integration

Travis CI Core Concepts for Beginners: A guide to the concept of continuous integration. https://docs.travis-ci.com/user/for-beginners/

Getting Started with Travis CI: Information about how to get started using Travis CI for your continuous integration needs https://docs.travis-ci.com/user/getting-started/

Getting Started with AppVeyor: AppVeyor is an alternative to Travis CI that can also be used for continuous integration, with GitHub or Bitbucket. https://www.appveyor.com/docs/

Getting Started with Jenkins: Jenkins is continuous integration that can be run on your own server. https://jenkins.io/doc/pipeline/tour/getting-started/

Jenkins learning resources: A compilation of web resources to help users with deploying Jenkins for continuous integration. https://dzone.com/articles/the-ultimate-jenkins-ci-resources-guide

Training

The Carpentries: Find a training workshop, or schedule your own, to train your team in all these tools, and more. https://carpentries.org/

Data Carpentry: http://www.datacarpentry.org/

Software Carpentry: https://software-carpentry.org/
